# Topical HPMC/S-Nitrosoglutathione Solution Decreases Inflammation and Bone Resorption in Experimental Periodontal Disease in Rats

**DOI:** 10.1371/journal.pone.0153716

**Published:** 2016-04-26

**Authors:** Conceição S. Martins, Renata F. C. Leitão, Deiziane V. S. Costa, Iracema M. Melo, Glaylton S. Santos, Vilma Lima, Victor Baldim, Deysi V. T. Wong, Luana E. Bonfim, Cíntia B. Melo, Marcelo G. de Oliveira, Gerly A. C. Brito

**Affiliations:** 1 Postgraduate Program in Morphofunctional Sciences, Department of Morphology, School of Medicine, Federal University of Ceará, Fortaleza, Ceará, Brazil; 2 Department of Clinical Dentistry, Faculty of Pharmacy, Dentistry and Nursing, School of Dentistry. Federal University of Ceará, Fortaleza, Ceará, Brazil; 3 Department of Physiology and Pharmacology, School of Medicine, Federal University of Ceará, Fortaleza, Ceará, Brazil; 4 Institute of Chemistry, University of Campinas, UNICAMP, Campinas, São Paulo, Brazil; Georgia Regents University, UNITED STATES

## Abstract

S-nitrosoglutathione (GSNO) is a nitric oxide (NO) donor, which exerts antioxidant, anti-inflammatory, and microbicidal actions. Intragingival application of GSNO was already shown to decrease alveolar bone loss, inflammation and oxidative stress in an experimental periodontal disease (EPD) model. In the present study, we evaluated the potential therapeutic effect of topical applications of hydroxypropylmethylcellulose (HPMC)/GSNO solutions on EPD in Wistar rats. EPD was induced by placing a sterilized nylon (3.0) thread ligature around the cervix of the second left upper molar of the animals, which received topical applications of a HPMC solutions containing GSNO 2 or 10 mM or vehicle (HPMC solution), 1 h prior to the placement of the ligature and then twice daily until sacrifice on day 11. Treatment with HPMC/GSNO 10 mM solution significantly reduced alveolar bone loss, oxidative stress and TNF-α e IL-1β levels in the surrounding gingival tissue, and led to a decreased transcription of RANK and TNF-α genes and elevated bone alkaline phosphatase, compared to the HPMC group. In conclusion, topical application of HPMC/GSNO solution is a potential treatment to reduce inflammation and bone loss in periodontal disease.

## Introduction

Periodontal diseases, including gingivitis, are highly prevalent [1] and characterized by the inflammation of the periodontal connective tissue with influx of inflammatory cells, especially polymorphonuclear leukocytes, monocytes and macrophages from the peripheral blood into the periodontal connective tissue. While gingivitis does not affect the underlying supporting structures of the teeth, periodontitis results in loss of connective tissue and bone support [2,3] and can progress to bone destruction, tooth mobility and finally, tooth loss. In turn, the bacterial pathogens in the dental plaque stimulate host cells to release various pro-inflammatory cytokines such as IL-1 and TNF-α, which attract polymorphonucleocytes (PMNs) to the site of infection. In addition, the activation of neutrophils by periodontopathogenic microorganisms with the consequent production of reactive oxygen species (ROS) may induce periodontal tissue breakdown [4].

Endogenously produced nitric oxide (NO) is a mediator of several physiological and pathophysiological processes whose actions include the inactivation of ROS and the blocking of radical propagation reactions [5,6]. It has also been demonstrated that NO has a variety of effects on bone, suppressing osteoclast bone resorption and promoting the growth of osteoblasts [7–13].

It has been demonstrated that intragingival administration of S-nitrosoglutathione (GSNO), an endogenously found NO carrier and donor decreases bone resorption *in vivo* in a animal model of periodontitis [14], similarly to what has been shown *in vitro* with L-arginine supplementation [15]. GSNO has already displayed microbicidal actions [16–18] as well as wound healing action [19] in several other studies. These results encouraged us to investigate the potential beneficial actions that could be obtained from the topical application of GSNO for treating periodontitis, avoiding thus an invasive approach. For this purpose, we incorporated GSNO in hydroxypropylmethylcellulose (HPMC) solution. HPMC is widely used as a hydrophilic pharmaceutical vehicle for drug delivery systems and due to its mucoadhesive property, comprises an adequate matrix for the topical NO delivery from GSNO in the oral cavity.

## Materials and Methods

### Reagents and drugs

Hydroxypropylmethylcellulose (HPMC; Mn 90000), glutathione (GSH), sodium nitrite (NaNO_2_), phosphate-buffered saline (PBS) pH 7.4, acetone and hydrochloric acid (HCl) were purchased from Sigma-Aldrich (St. Louis, MO, USA). All reagents were analytical grade and used as received. All experiments were performed using deionized water from a Millipore Milli-Q gradient filtration system (Billerica, MA, USA).

### S-nitrosoglutathione synthesis

S-nitrosoglutathione (GSNO) was synthesized by reacting GSH with NaNO_2_ in equimolar amounts in acidic solution (HCl, pH 2.0) as described elsewhere [19]. Solid, dry GSNO was stored protected from light at -20°C for further use.

### Preparation of HPMC/GSNO solutions

To incorporate GSNO in the HPMC solutions at final concentrations of 2.0 mM (HPMC/GSNO 2.0 mM) and 10 mM (HPMC/GSNO 10 mM), appropriate amounts of solid GSNO were weighted and transferred initially to PBS solution in falcon tubes. After complete GSNO dissolution under stirring, appropriate volumes of the GSNO solutions were transferred to other falcon tubes containing solid HPMC in amounts calculated to obtain a final HPMC concentration of 1.25 wt% in all cases. The tubes were stirred for 5 min in a vortex and transferred to a refrigerator at 8°C, where they stayed for 2 h to assure complete dissolution of the HPMC in the GSNO solutions. The GSNO solutions and HPMC/GSNO solutions were protected from room light with aluminum foil during all the procedures in order to avoid GSNO photodecomposition. The prepared formulations were used immediately in the topical applications and stored back in the refrigerator after use. The formulations were discarded after 3 days of storage.

### Animals

Male Wistar rats from the Federal University of Ceará, weighting 200 to 250 g were housed in temperature-controlled rooms under 12 hour light-dark cycles and received water and food *ad libitum*. Surgical procedures and animal treatments were conducted in accordance with the guidelines of the Animal Research Ethics Committee (Comissão de Ética em Pesquisa Animal—CEPA/DFF/FAMED/UFC—Permit number: 10/2013), of the Federal University of Ceará, Brazil, who approved all the procedures involving the animals. All efforts were made to minimize suffering.

### Experimental Periodontal Disease Protocol

Experimental Periodontal Disease (EPD) was induced as previously described [20–21].Briefly, the animals were anesthetized with ketamine (90 mg/kg intraperitoneally) and xylazine (10 mg/kg intraperitoneally) and a sterilized nylon (0.3) thread ligature was placed around the cervix of the second left maxillary molar. The ligature was then knotted on the vestibular side of the tooth, so it remained subgingival on the palatal side and supragingival on the buccal side. The animals were euthanized with an overdose of ketamine and xylazine (300/30mg/kg; i.p.) on day 11^th^.

### Topical application of HPMC/GSNO solutions

The animals were divided into groups of 6 animals each and received topical applications of HPMC/GSNO 2.0 mM or HPMC/GSNO 10.0 mM. The solutions were applied using small cotton swabs with a gentle massage to both palatal and vestibular sides of the left maxillary molars gingiva, starting 1 hour before the placement of the ligature and twice daily until euthanasia on the 11^th^ day. Control groups consisted of animals subjected to the surgical procedure that received only the vehicle (HPMC) and a group of animals not subjected to experimental periodontitis (normal).

### Alveolar Bone Loss

The animals were euthanized 11 days after the placement of the ligature and the maxillae were excided and fixed in 10% buffer formalin. After 24 hour, the left maxillae were defleshed and stained with methylene blue (1%) to differentiate bone from teeth, fixed in a piece of wax and photographed. The digital images were subjected to alveolar bone loss analysis using the Image J software, as described previously [22]. In this procedure, the area (mm^2^) corresponding to the exposed roots of the molars (no bone covering them at all) was calculated and subtracted from the corresponding area (mm^2^) of the normal group. All acquired images were compared to a well-known area (1.0 x 1.0 mm^2^).

### Histopathological Analysis

For the histopathological analysis, the maxillae were excised, fixed in 10% neutral-buffered formalin and demineralized in EDTA. The specimens were then dehydrated, embedded in paraffin, and sectioned along the molars in a mesiodistal plane for hematoxylin and eosin staining. Three sections of 4 μm each, corresponding to the area between the first and second molars were evaluated under light microscopy using a 0 to 3 score grade [21,23], as following: 0- absence or discrete cellular infiltration (inflammatory cell infiltration is space and restricted to the region of the marginal gingival) and preserved alveolar process and cementum; 1- moderate cellular infiltration (inflammatory cellular infiltration present all over the insert gingival), some but discrete alveolar process resorption and intact cementum; 2- Accentuated cellular infiltration (inflammatory cellular infiltration present in gingival and periodontal ligament), moderate degradation of the alveolar process and partial destruction of cementum; 3- Accentuated cellular infiltrate, complete resorption of the alveolar process and severe destruction of cementum.

### Plasma Bone Alkaline Phosphatase (BALP)

Blood samples were collected from retro-orbital plexus, under anesthesia with a mixture of ketamine and xylazine (90/10 mg/kg; i.p.), before ligature (on day 0) and immediately before the euthanasia on the 11^th^ day in order to obtain plasma concentration of alkaline phosphatase using the thermo-activation method, as previously described [24]. The samples were heated up to 56°C for 10 minutes. Serum levels of BALP (a thermosensitive isoform of the total alkaline phosphatase, TALP), were calculated by subtracting the concentration of the heated alkaline phosphatase in serum from the concentration of TALP in serum. The analysis was performed according to the manufacturer’s instructions (Labest^®^, Lagoa Santa, MG, Brazil). The results were expressed as Bone Alkaline Phosphatase variation on day 11 (U/L) compared to the values obtained before the EPD induction, on day 0.

### Malonaldehyde and Reduced Glutathione (GSH) Gingival Levels

To evaluate the effect of HPMC/GSNO solutions on the oxidative stress, reduced glutathione (GSH), an antioxidant and malonaldehyde, a marker for oxidative stress, were measured in the gingival tissue collected on day 11. Lipid peroxidation was measured as malonaldehyde (MDA) production through the thiobarbituric acid reaction [25] in the gingival tissue of rats. Briefly, 250 μl of 10% homogenate of gingival tissue were mixed with 1.5 ml of 1% H_3_PO_4_ and 0.5 ml of 0.6% thiobarbituric acid aqueous solution and the mixture was stirred and heated in boiling water for 45 minutes. After cooling, 2 ml of n-butanol were added and the mixture was homogenized. The butanol layer was separated, and the difference between the optical densities at 535 and 520 nm was used for calculating the MDA concentrations, which were expressed as nanomol of MDA per gram of gingival tissue.

To evaluate the reduced glutathione (GSH) levels, the gingivae were first homogenized (400 μl of a 10 wt% tissue solution prepared in 0.02 M EDTA). After that, 320 μl of distilled water and 80 μL of 50% trichloroacetic acid (TCA) were added, followed by centrifugation at 3000 rpm for 15 min at 4°C. The supernatant was collected (400 mL) and added to 800 μL of 0.4 M Tris buffer at pH 8.9 and 20 μL of 0.01 M DTNB. The absorbance of each sample was measured at 412 nm, and the results were described as μg of GSH per milligram of tissue.

### Nitrite/nitrate (NO_x_) Gingival Levels

On day 11 after EPD induction, the gingivae surrounding the upper left molars were removed for nitrite/nitrate (NO_X_) quantification using the Griess method [26]. Firstly, nitrate was reduced to nitrite by incubation with nitrate reductase and nicotinamide adenine dinucleotide. Then, the Griess reagent (1% sulfanilamide and 0.1% naphthyl ethylenediamine dihydrochloride in 5% phosphoric acid) was added, and the total concentration of nitrite was evaluated through its absorbance at 540 nm, assigned to the Griess reaction adduct. A calibration curve was obtained by incubating sodium nitrite (10 to 200 μM) with the Griess reagent. Nitrate/nitrite levels were expressed as micromole per milligram of gingival tissue.

### Quantification of Cytokines Gingival Levels

The gingivae surrounding the upper left molars were removed on the 11^th^ day for and Tumor Necrosis Factor Alpha and Interleukin-1β (TNF-α and IL-β; DuoSet Elisa Development kit, R&D Systems, Minneapolis, MN, USA) measurements. The quantification of the cytokines contained in the samples was performed using an enzyme-linked immunosorbent assay (ELISA), as previously described [27,28]. TNF-α and IL-β levels in the gingival tissue were determined using respective standard curves. The results are expressed as pg/ml of TNF-α or IL-β.

### Immunohistochemical staining for inducible nitric oxide synthase (iNOS), receptor activator of the nuclear factor kappa-B (RANK) and Tartrate-resistant acid phosphatase (TRAP) and nitrotyrosine

After the animals were sacrificed on the 11^th^ day, the maxillae were excised, fixed in 10% neutral-buffered formalin and demineralized in 10% EDTA. The immunohistochemical for iNOS, RANK and TRAP were performed using the avidin–biotin–peroxidase method [27,28] in formalin-fixed, paraffin-embedded tissue sections (4 μm thick) and mounted on poly-L-lysine-coated microscope slides. The sections were deparaffinized and rehydrated through xylene and graded alcohols. After antigen retrieval, endogenous peroxidase was blocked (30 minutes) with 3% (v/v) hydrogen peroxide and washed in phosphate-buffered saline (PBS). Sections were incubated overnight (4°C) with primary polyclonal rabbit anti-iNOS antibody (1:200), primary polyclonal rabbit anti-RANK (1:200) and primary polyclonal rabbit anti-TRAP (1:200) or primary mouse monoclonal anti-nitrotyrosine (1:200). All the antibodies were diluted in PBS plus bovine serum albumin (BSA). The slides were then incubated with biotinylated goat anti-rabbit diluted 1:200 in PBS–BSA. After washing, the slides were incubated with avidin–biotin–horseradish peroxidase conjugate for 30 min, according to the protocol of the manufacturer. iNOS, RANK and TRAP and nitrotyrosine were visualized with the chromogen 3,3 diaminobenzidine (DAB), after 2 minutes of incubation. Negative control sections were processed simultaneously as described above but with the first antibody being replaced by 5% PBS–BSA. Slides were counterstained with hematoxylin, dehydrated in a graded alcohol series, cleared in xylene, and coverslipped. The quantitative estimation of DAB products from immunostaining was determined from digital images of at least ten different areas of each section (from four specimens per group), on 400x magnification, using Photoshop software. The immunostaining was calculated dividing the DAB positive staining (immunostaining positive pixels) by the number of pixels per image, as previously described [29].

### Quantitative PCR analysis of TNF-α, RANK and RANKL/OPG mRNA levels

Expression of mRNA for TNF-α, RANK, RANK-L and OPG was determined by quantitative real-time polymerase chain reaction (qPCR). RNA was extracted from gingival tissue using the *Aurum*^*TM*^ *Total RNA Fatty and Fibrous Tissue Kit* (Bio-Rad, CA, USA) following the manufacturer’s instructions. The quality of the RNA was analyzed by 260/280 ratio, and quantified by UV absorption using NanoDrop (Thermo scientific, Wilmington, DE, USA). One microgram of total RNA from the intestinal samples in a final volume of 20 μl were reverse-transcribed into cDNA in the C1000 Touch^™^ Termal Cycler system with the iScript^™^ cDNA synthesis kit from Bio-Rad. Real-time quantitative PCR analysis of the mRNA was performed in an CFX96 Touch^™^ real-time PCR detection system instrument from Bio-Rad using the iQTM SYBR^®^ Green Supermix (Bio-Rad, CA, USA) as indicated by the manufacturer. qPCR was performed for 35 cycles under the following conditions: denaturation 95°C, 30 s; annealing 60°C, 30 s; extension, 25°C, 50 s. All samples were run in duplicate, and the relative mRNA expression level was determined after normalizing all values to those of GAPDH. All samples were evaluated for the dissociation characteristics of the double-stranded DNA during heating (melting curve analysis). The relative gene expression was determined using the 2^-ΔΔCt^ method [30] with GAPDH as the housekeeping gene. The primer pairs used in this study are shown in Table 1.

**Table 1:** Description of biomarkers, gene, primer sequences and NCBI accession numbers.

Biomarkers	Gene	Primer sequence (5’-3’)	NCBI
Proinflammatory cytokines	TNF-α	**S-**GACCCTCACACTCAGATCATCTTCT **A-**TGCTACGACGTGGGCTACG	NM-012675
Bone markers	RANK	**S-**AGGGAAAACGCTGACAGCTAA **A-** CCAACACAATGGTCCCCTGA	NM_001271235
	RANK-L	**S-**GCCAACCGAGACTACGGCAA **A-**GAACATGAAGCGGGAGGCG	NM_057149
	OPG	**S-**CCTAGAGAGAAATGTCGTAGGATT **A-**CATTCCACACTGGAAACCTGA	NM_012870
Reference gene	GAPDH	**S-**TGATTCTACCCACGGAAGTT **A-**TGATGGGTTTCCCATTGATGA	NM_017008

### Statistical Analysis

Data are presented as means ± standard error (SEM) or as medians when appropriate. Univariate Analysis of Variance followed by Bonferroni test was used to compare means and Kruskal–Wallis and Dunn tests were used to compare medians. P< 0.05 was considered significant. All analyses were performed using GraphPad Prism 6 software, San Diego, CA, USA.

## Results

### Alveolar Bone Loss

**Fig 1:**
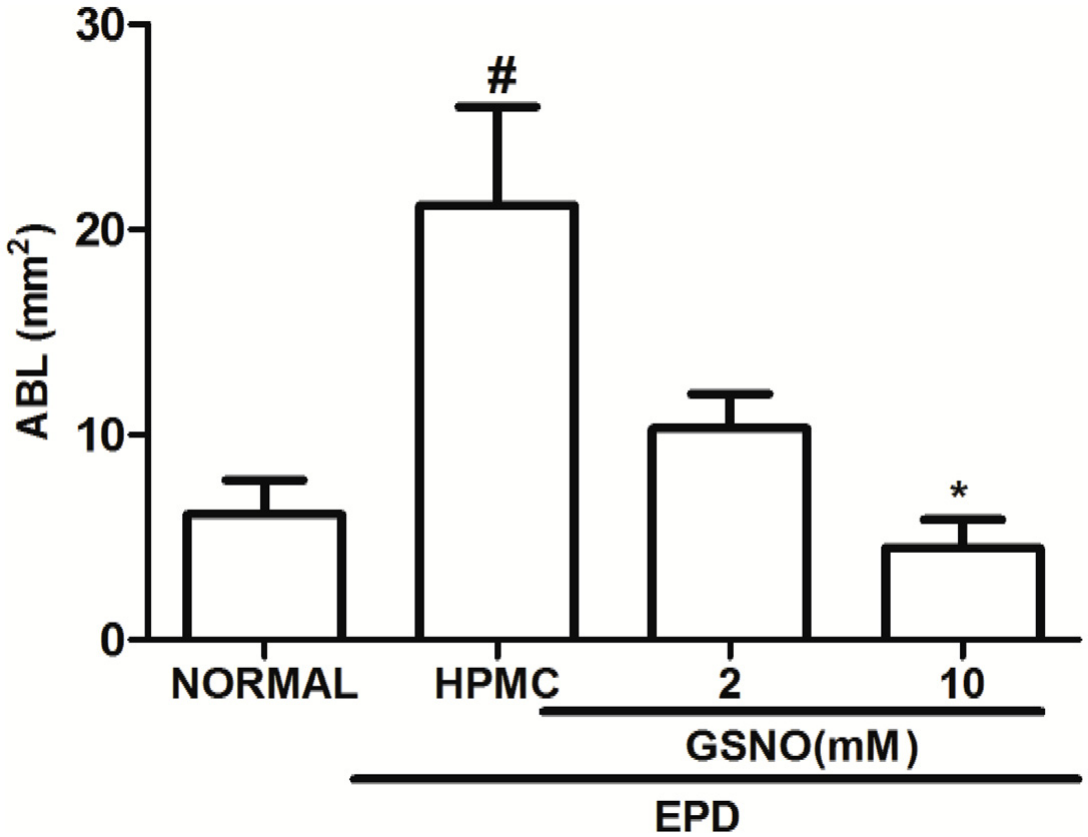
Effect of topical application of HPMC/GSNO solutions on alveolar bone loss in experimental periodontitis. Data represent the mean ± SEM of six animals in each group. #P<0.05 was considered significantly different compared to the normal control group (NORMAL); *P<0.05 was considered significantly different compared to rats subjected to experimental periodontitis, which received topical application of the vehicle (HPMC). (ANOVA; Bonferroni’s test).

**Fig 2:**
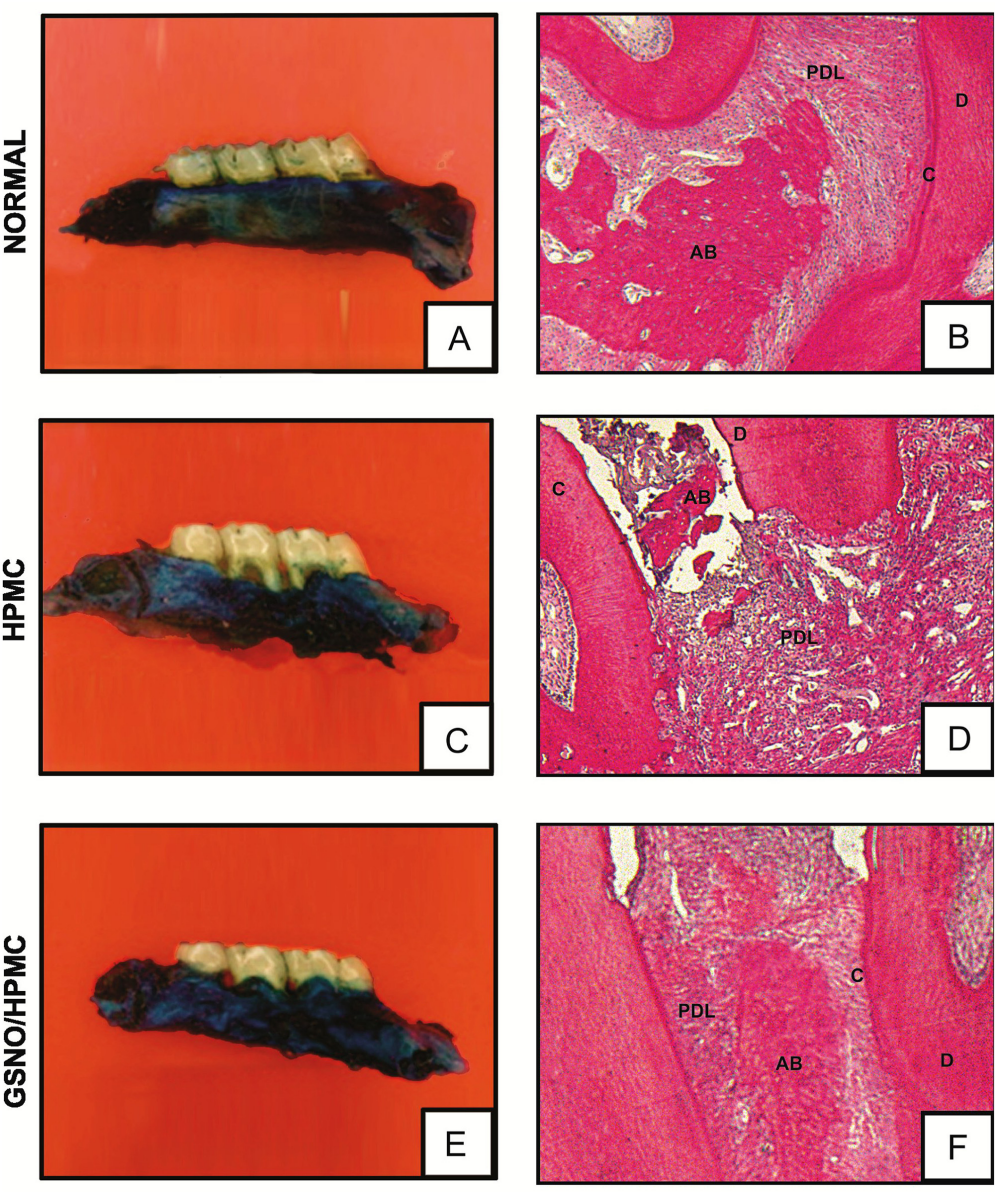
**Effect of topical application of HPMC/GSNO solutions on the macroscopic (first column) and histological (second column) aspects of periodontium (A and B)** normal maxilla, showing integrity of its components (c. Cementum, pl. Periodontal ligament, ab. Alveolar bone and g. Gingiva). (**C and D)** Maxilla subjected to experimental periodontitis that received just the vehicle (HPMC), showing severe bone resorption, inflammatory infiltrate in both the gingiva and periodontal ligament, extensive cementum destruction and total resorption of the alveolar process, (**E and F)** Maxilla after 11 days of experimental periodontitis treated with 2.0 mM HPMC/GSNO showing discrete cell influx and preservation of the alveolar process and cementum. Picrosirius staining (magnification x40).

Local administration of HPMC/GSNO 10 mM solution 1 hour before the placement of the ligature and twice daily for 11 days resulted in a significant inhibition of alveolar bone loss (Figs 1 and 2E), compared to the hemimaxillae of animal groups that received the vehicle only (HPMC; Figs 1 and 2C). No significant bone loss was observed in the normal groups (Figs 1 and 2A).

Histopathological analysis of the region between the first and second molars of the animal periodontium that received local applications of HPMC demonstrated accentuated inflammatory leukocyte infiltration, resorption of the alveolar bone and cementum and collagen fiber derangement within the periodontal ligament, receiving a median score and range of 3(2–3) (Table 2; Fig 2D). The periodontium of rats treated with the HPMC/GSNO 10 mM solution showed preservation of the alveolar process and cementum, reduction of the inflammatory cell infiltration and partial preservation of the collagen fibers of the periodontal ligament, receiving a median score and range of 1(0–2) (Table 2; Fig 2F). The histological analysis of the region between the first and second molars of the control group that was not subjected to EPD (Normal), shows the structures of the normal periodontium, where fingiva (G), periodontal ligament (PDL), alveolar bone (AB), cementum (C) and dentin (D) can be observed [(0(0–0)] (Table 2; Fig 2B).

**Table 2:** Effect of topical application of GSNO/HPMC formulation on histopathologic score of rat maxillae.

Groups	Scores (average values and range)
**CONTROL**	0 (0–0)
HPMC^1^	3 (3–3)#
GSNO (0.5 mM)^1^	2,5 (2–3)
GSNO (2 mM)^1^	2,5 (2–3)
GSNO (10 mM)^1^	1,5 (1–2)*

EPD: Experimental periodontal disease.

^1^ Animals submitted to EDP;

^#^ p< 0.05 versus the normal controls (CONTROL);

* p< 0.05 versus HPMC (animals submitted to EDP and treated with vehicle).

### Plasma Bone Alkaline Phosphatase (BALP)

**Fig 3:**
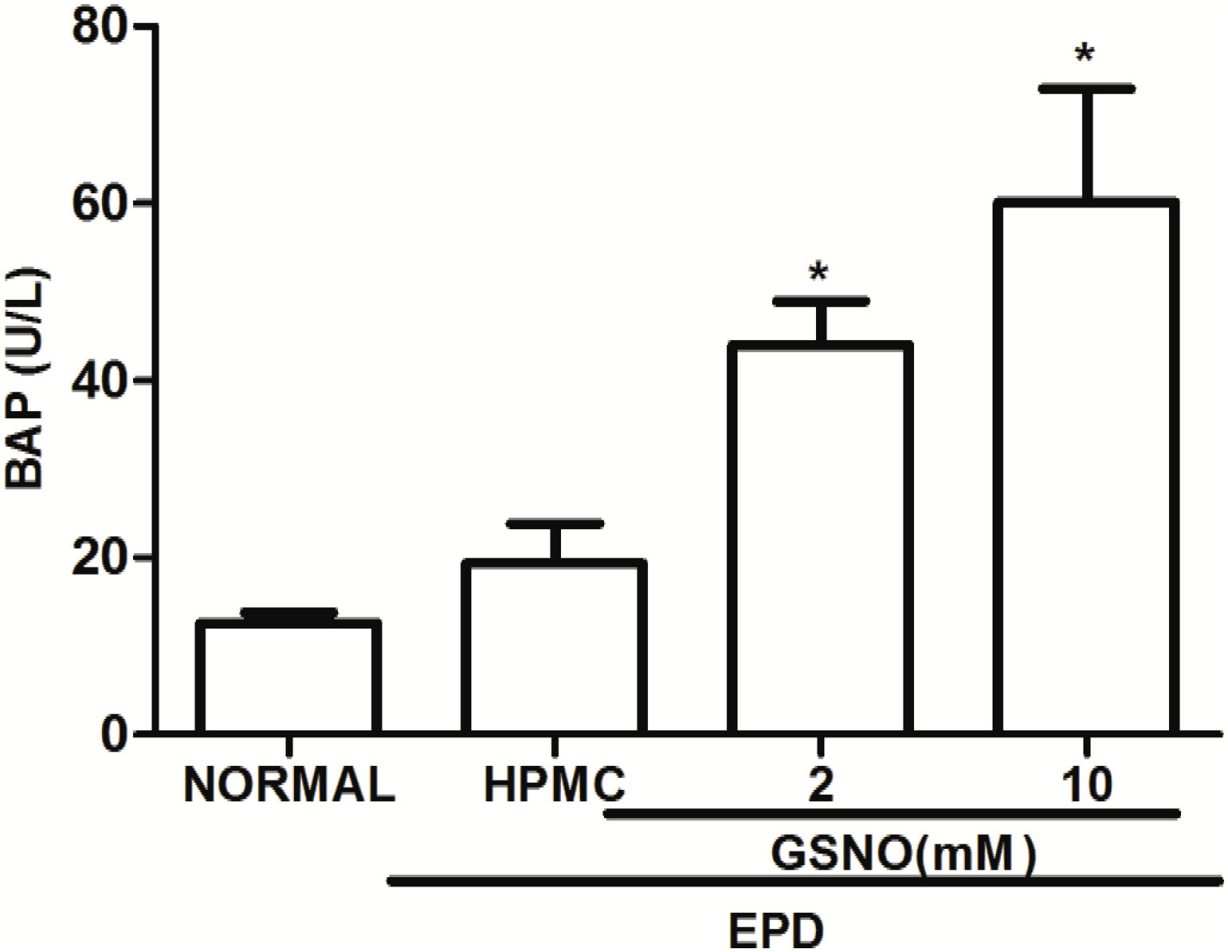
Effect of topical application of HPMC/GSNO solutions on the plasma bone alkaline phosphatase (BAP) in experimental periodontitis in rats. Data represent the mean ± SEM of six animals for each group. #P<0.05 was considered significantly different compared to the normal control (NORMAL) and to the group of animals subjected to experimental periodontitis that received topical applications of the vehicle (HPMC). Analysis of variance (ANOVA); Bonferroni test.

The treatment with GSNO/HPMC (2 and 10 mM) resulted in a significant increase in the BALP serum levels variation when compared with the control HPMC solution group (Fig 3).

### Malonaldehyde (MDA) and Glutathione (GSH) Gingival levels

**Fig 4:**
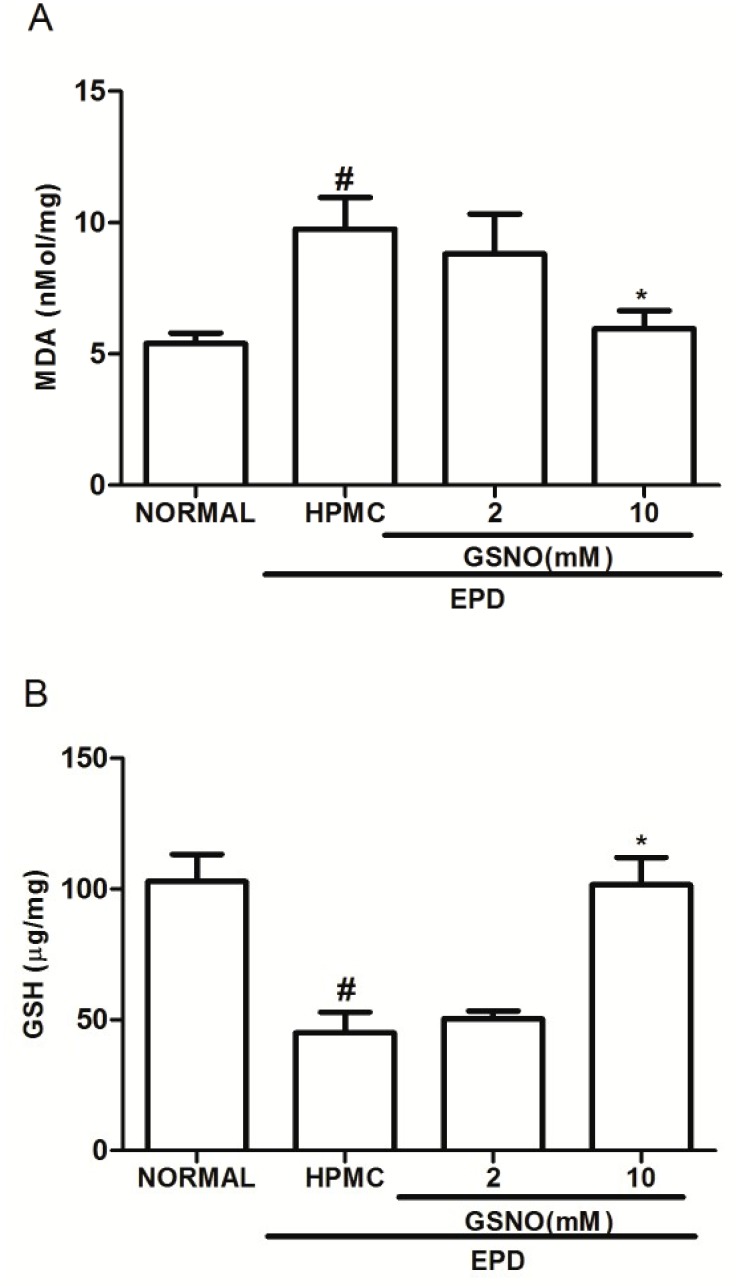
Effect of the topical application of HPMC/GSNO solutions on the oxidative stress markers malonaldehyde (MDA; A) and reduced glutathione (GSH; B) in experimental periodontitis in rats. Data represent the mean ± SEM of six animals for each group. #P<0.05 was considered significantly different compared to the normal control (NORMAL); *P<0.05 was considered significantly different compared to the group of animals subjected to experimental periodontitis that received topical applications of the vehicle (HPMC solution). Analysis of variance (ANOVA); Bonferroni test.

At the 11^th^ day the group subjected to EPD that received topical applications of the control HPMC solution showed a significant increase in the MDA level and a reduction in the GSH concentration in the gingival tissue, when compared to the normal group. At the same time, treatment with GSNO/HPMC at 10 mM (but not 2.0 mM) significantly reduced the MDA gingival tissue levels (Fig 4A) and increased GSH levels (Fig 4B) when compared with HPMC group.

### Nitrite/Nitrate (NOx) Gingival Levels

**Fig 5:**
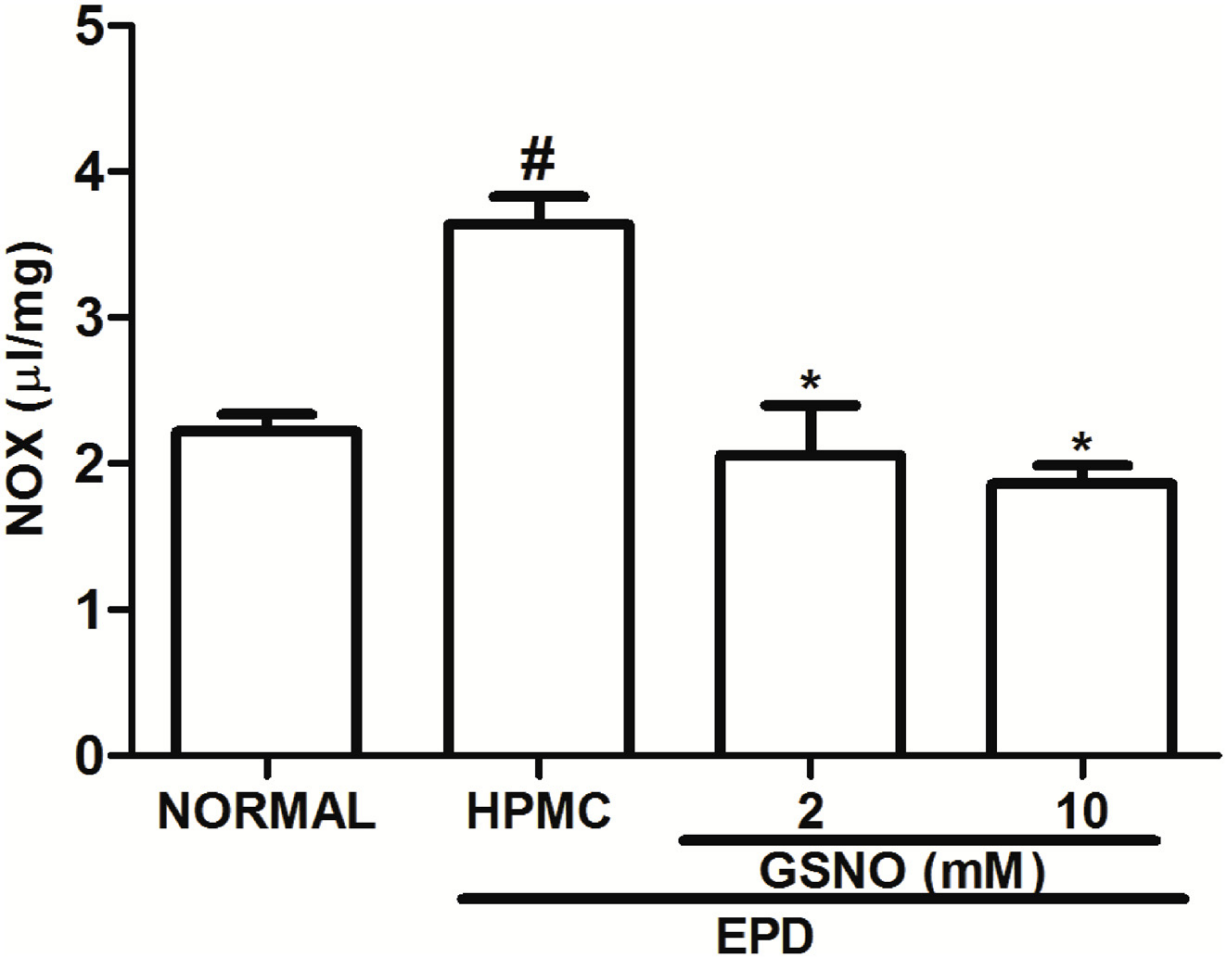
Effect of HPMC/GSNO on the nitrite/nitrate (NOX) levels in experimental periodontitis in rats. Data represent the mean ± SEM of six animals for each group. # P<0.05 was considered significantly different compared to the normal control (NORMAL); *P<0.05 was considered significantly different compared to the group of animals subjected to experimental periodontitis that received topical applications of the vehicle (HPMC). Analysis of variance (ANOVA); Bonferroni test.

The group subjected to EPD that received topical applications of the control HPMC solution showed a significant increase in NOx levels when compared to the Normal group, on day 11. Treatment with the HPMC/GSNO solutions (2 and 10 mM) significantly reduced NOx concentration when compared with the control HPMC solution group (Fig 5).

### Gingival Tissue cytokine levels

**Fig 6:**
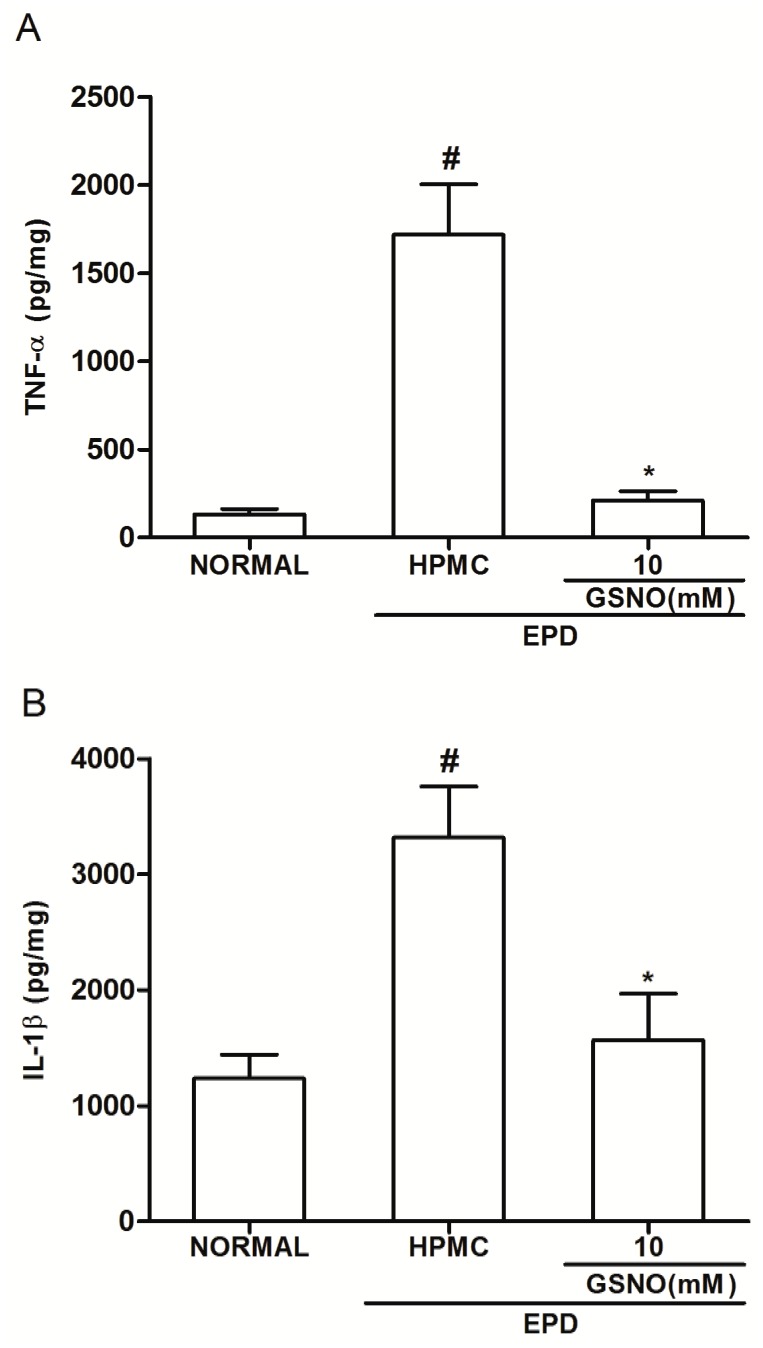
Effect of HPMC/GSNO on tumor necrosis factor alpha (TNF-α; A) and interleukin-1β (IL-1β; B) in experimental periodontitis in rats. Data represent the mean ± SEM of six animals for each group. # P<0.05 was considered significantly different compared to the normal control (NORMAL); *P<0.05 was considered significantly different compared to the group of animals subjected to experimental periodontitis that received topical applications of the vehicle (HPMC). Analysis of variance (ANOVA); Bonferroni test.

It was observed a significant increase in the TNF-α (Fig 6A) and IL-1β (Fig 6B) levels in the gingival tissue of animals subjected to EPD that received topical applications of control HPMC solution, when compared with the Normal control group. Topical treatment with the HPMC/GSNO 10 mM solution significantly reduced cytokines levels when compared with the control HPMC solution group (Fig 6A and 6B).

### Immunohistochemical staining for iNOS, RANK and TRAP and nitrotyrosine

**Fig 7:**
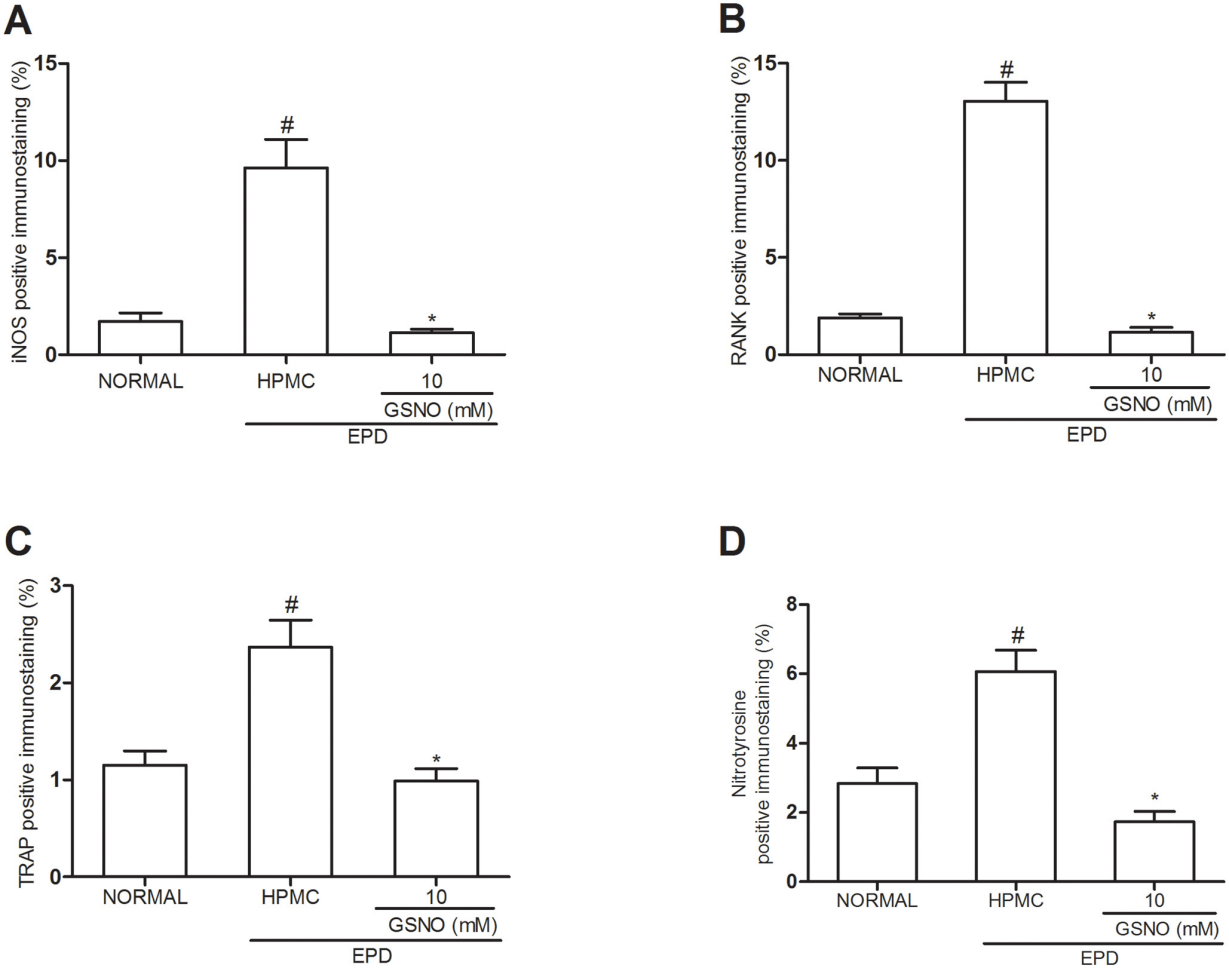
Quantification of iNOS, RANK, TRAP and nitrotyrosine immunostainning in periodontal tissue of rats submitted to experimental periodontitis. Data represent the mean ± SEM of four animals for each group. # P<0.05 was considered significantly different compared to the normal control (NORMAL); *P<0.05 was considered significantly different compared to the group of animals subjected to experimental periodontitis that received topical applications of the vehicle (HPMC). Analysis of variance (ANOVA); Bonferroni test.

**Fig 8:**
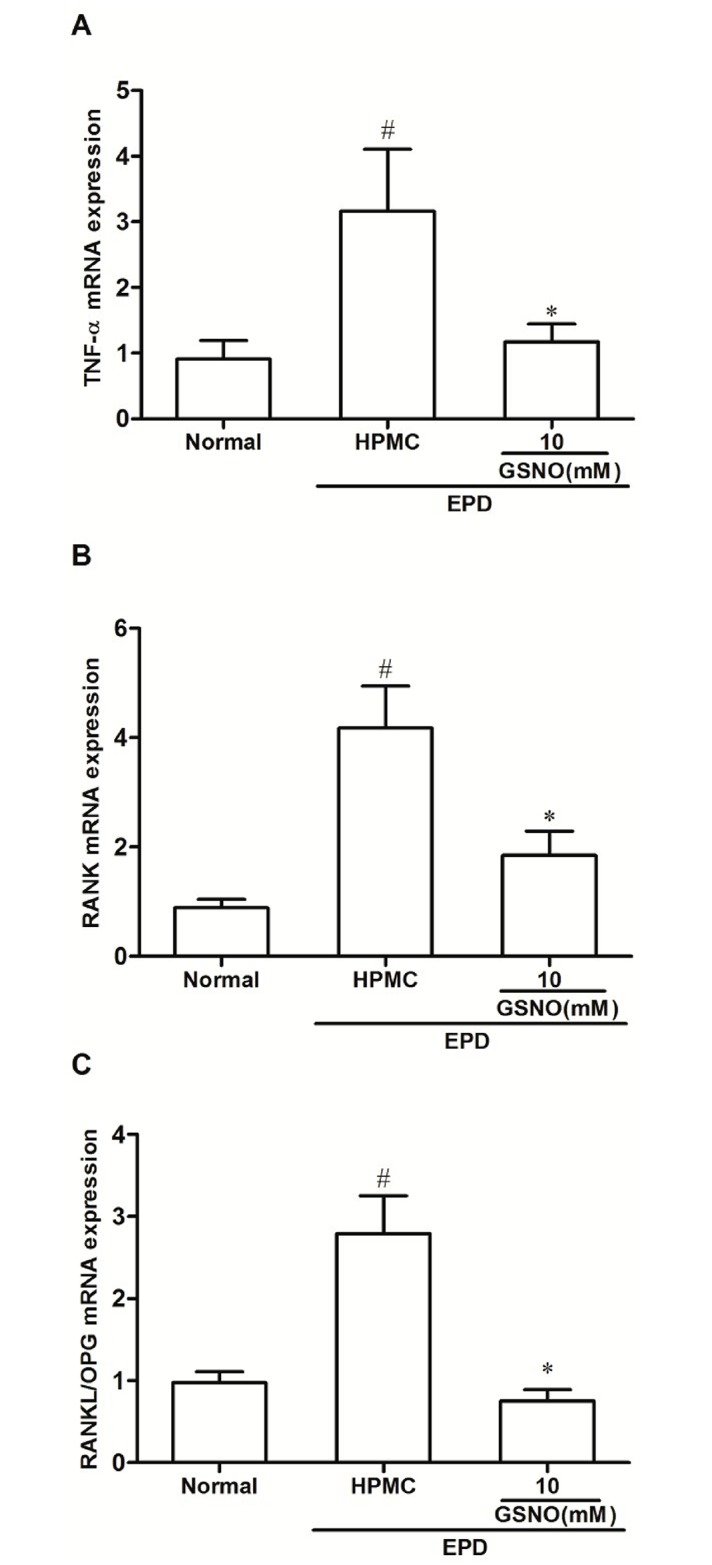
Representative examples of iNOS (1^st^ row), RANK (2^nd^ row) and TRAP (3^rd^ row) immunostainning in experimental periodontitis in rats. Staining was performed using periodontal tissues from normal control animals (b, g, l, q), animals subjected to experimental periodontitis that received topical applications of HPMC (c, h, m, r) or 10 mM HPMC/GSNO (d, i, n, s). Negative controls were samples of periodontal tissue where the primary antibody was replaced with PBS-BSA (5%); no immunostaining was detected (a, f, k, p). Magnification x200. Arrows points to immunostaining osteoclasts in the periodontal tissue of the control HPMC solution group (Magnification x1000).

Fig 7 illustrates immunostaining for iNOS (Fig 7A), RANK (Fig 7B) and TRAP (Fig 7C) and nitrotyrosine (Fig 7D) immune expression in the periodontal tissue of animals subjected to EPD that received topical applications of control HPMC solution, when compared with the Normal control group. Topical treatment with the HPMC/GSNO 10 mM solution significantly reduced the immunostaining of iNOS, RANK, TRAP and nitrotyrosine, simultaneously, when compared with the control HPMC solution group. Accordingly, Fig 8 illustrates immunostaining for iNOS, RANK, TRAP and nitrotyrosine in the periodontal tissue of animals subjected to EPD that received local applications of the control HPMC solution (Fig 8C, 8H, 8M and 8R respectively) on the 11^th^ day compared with the weak staining observed in the Normal control group (Fig 8B, 8G, 8L and 8Q respectively). Local treatment with the HPMC/GSNO 10 mM solution for 11 days resulted in a considerable reduction in iNOS, RANK and TRAP immunostaining (Fig 8D, 8I, 8N e 8S respectively) when compared with the control HPMC solution group (Fig 8C, 8H, 8M and 8R, respectively). When the antibodies were replaced with PBS/BSA 5%, no immunostaining was detected (negative controls; Fig 8A, 8F and 8K and 8P). Fig 8E, 8J, 8O and 8T illustrate the strong immunostaining observed in the osteoclasts in the control HPMC solution group.

### Quantitative PCR analysis of RANK, TNF-α and RANKL/OPG mRNA levels

**Fig 9:**
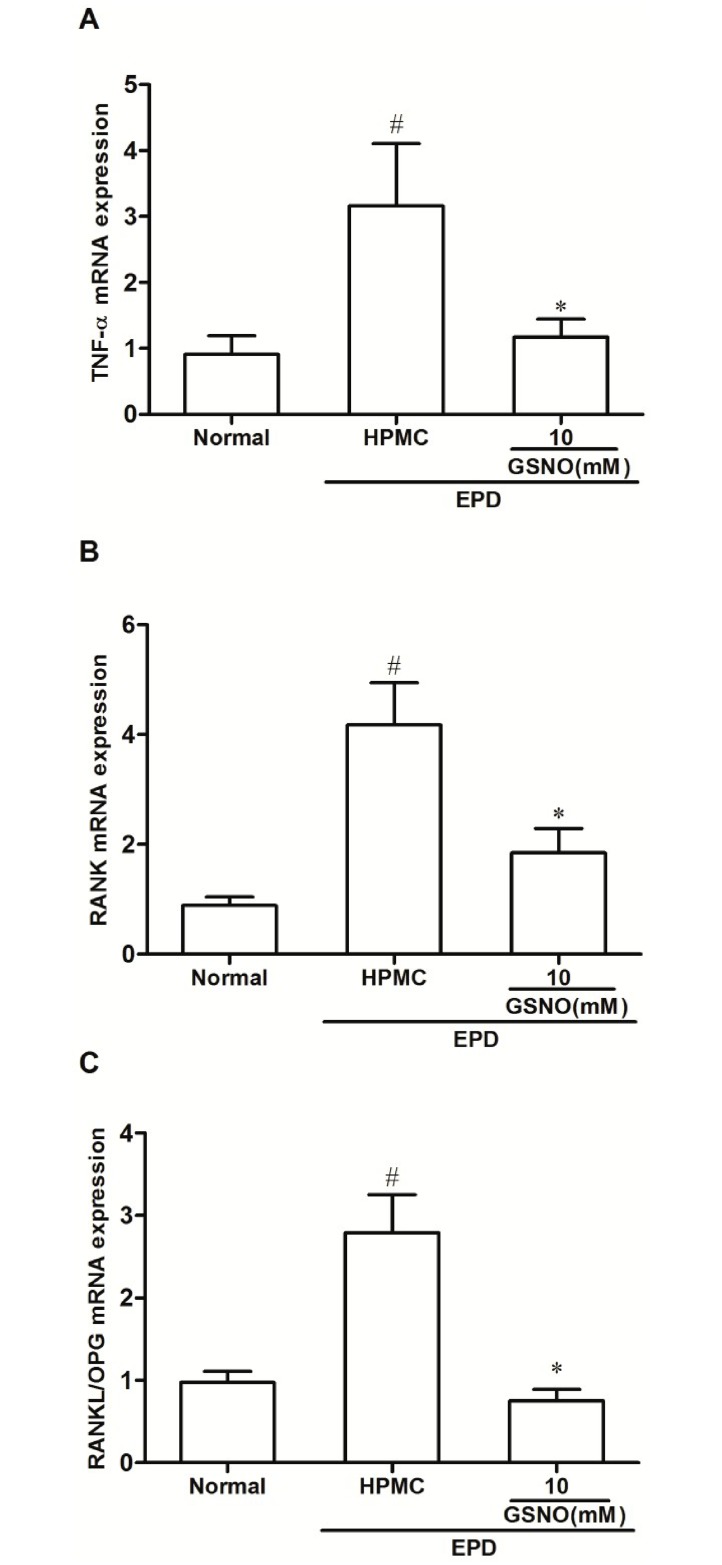
Effect of HPMC/GSNO on tumor necrosis factor alpha (TNF-α; A), RANK (B) and on the ratio RANKL/OPG (C) mRNA levels in experimental periodontitis in rats. Data represent the mean ± SEM of six animals for each group. # P<0.05 was considered significantly different compared to the normal control (NORMAL); *P<0.05 was considered significantly different compared to the group of animals subjected to experimental periodontitis that received topical applications of the vehicle (HPMC). Analysis of variance (ANOVA); Bonferroni test.

The group subjected to EPD that received local applications of the control HPMC solution showed a significant increase in the TNF-α (Fig 9A), RANK (Fig 9B) and in the ratio RANKL/OPG (Fig 9C) mRNA expression when compared with the Normal control group. Topical treatment with the HPMC/GSNO 10 mM solution prevented the increase in all cases (Fig 9A, 9B and 9C).

## Discussion

In the present study, we demonstrated that topical application of HPMC solution containing GSNO 10 mM reduces the alveolar bone loss associated with EPD when compared with the control group that received only the HPMC solution. This result is in accordance with previous studies demonstrating that both GSNO and S-nitroso-N-acetylpenicilamine (SNAP) reduce inflammation and bone loss in EPD [11,14]. The absence of anti-reabsorptive or anti-inflammatory effects of topical applications of the HPMC/GSNO 2 mM solution suggests that this GSNO concentration is insufficient to provide a beneficial effect in the present EPD model. This result is consistent with the literature, which shows that the biological effect of exogenous NO administration is highly dependent on its local concentration [11,14,31]. HPMC/GSNO solutions were already shown to release free NO from the spontaneous thermal decomposition of GSNO according to the equation:

2 GSNO →  GS−SG+2 NO (1)

where GS-SG is oxidized glutathione, formed after the homolytic S-N bond cleavage, followed by the formation of a covalent S-S bond [14,19,32]. Free NO locally released at the HPMC/GSNO solution/gingiva interface is expected to diffuse through the gingiva and periodontal tissues and to be the chemical species responsible for the biological actions herein reported.

In the present HPMC/GSNO solutions the HPMC concentration is 1.25 wt%. At this concentration, the HPMC chains are considered to be fully hydrated, with little polymer-polymer interactions, comprising thus a viscous polymeric solution and not a hydrogel. HPMC solutions can undergo gelation with HPMC concentrations above 10 wt% and HPMC hydrogels are frequently used as vehicles for drug delivery [33,34]. The lower HPMC concentration used in the present study was chosen to facilitate the spreading of the formulation on the affected gingiva, avoiding thus any inflammation that could be caused by the topical of a hard hydrogel.

The observed therapeutic effect of the HPMC/GSNO 10 mM solution on the bone resorption was associated with an increase in the level of plasma bone alkaline phosphatase (BALP), a marker of osteoblastic activity, in the serum of animals and a considerable reduction in TRAP immunostaining (a histochemical marker of osteoclasts observed in multinuclear cells, mainly osteoclast). These changes were observed in the periodontal tissue of the animals treated with he HPMC/GSNO 10 mM solution for 11 days, when compared with the control HPMC solution group, suggesting that the HPMC/GSNO 10 mM solution prevents bone resorption and also induces bone formation. Accordingly, it has been described that NO donors have beneficial effects on the control of bone resorption (decreased osteoclast activity), and have a milder anabolic action on bone formation, enhancing osteoblast activity [12,13].

The present study suggests that the protective effect of GSNO on the alveolar bone resorption is at least in part due to its anti-inflammatory, anti-oxidant and immunomodulatory properties. We demonstrated that the HPMC/GSNO 10 mM solution significantly reduced the TNF-α mRNA in gingival tissue of animals treated with this formulation as well as the gingival levels of TNF-α and IL-1β, when compared with the control HPMC solution group.

Cytokine participation in periodontal disease is well documented and these substances, particularly TNF-α and IL-1β, may amplify the inflammatory response causing tissue destruction and bone loss [31]. It has been reported that IL-1 and TNF-α activate the transcription of inducible nitric oxide synthase (iNOS) gene resulting in NO production [26]. The HPMC/GSNO 10 mM solution markedly reduced both iNOS and nitrotyrosine expressions in the periodontal tissues and also reduced the NOx levels in the gingival tissues in comparison with the control HPMC solution. These findings are in accordance with those reported by Menezes et al. (2012) who showed that GSNO significantly reduces the gingival levels of IL-1, TNF-α and NOx, associated with the reduction of iNOS expression in a study conducted using the same experimental model of the present work [14]. The reduction of iNOS expression associated with the administration of exogenous GSNO was also described in other studies, notably in the protective action against secondary injury in an animal model of traumatic brain injury [35]. This effect has been attributed to the S-nitrosation of the p65 subunit of nuclear factor kappa B (NFκB) by GSNO, via transnitrosation reaction in the endothelial cells, with consequent inhibition of NFκB activation and down regulation of iNOS. Alternatively, or concomitantly, the mechanism may involve the negative feedback modulation of iNOS by NO itself, including self-generated and exogenous NO [36]. In this case, the mechanism is considered to involve the coordination of NO to the iron center of the heme group of iNOS, and can be extended to other NOS isoforms [37]. The buildup of Heme-NO complex is expected to strongly inhibit the catalytic activity of the NOSs. In the present case, the source of NO for this negative feedback action on iNOS, could be attributed to the exogenous NO locally released by the HPMC/GSNO formulation, according to Eq 1.

The release of large amounts of NO by iNOS has been shown to play a major role in immune reactions and inflammatory processes, including rheumatoid arthritis and periodontal disease [23]. NO reacts with oxygen-derived free radicals such as superoxide anion to form highly reactive molecules such as peroxynitrite anion and the hydroxyl radical and production of such species may contribute to tissue damage by inducing lipid peroxidation [38]. In accordance, we demonstrated that EPD leads to an oxidative stress in periodontal tissue with consequent generation of reactive oxygen species, indicated by the significant increase in MDA levels associated with an increase in the nitrotyrosine expression in the periodontal tissue and a decrease in GSH levels in the gingival tissue. Topical application of HMPC/GSNO 10 mM solution attenuated the gingival oxidative stress, reestablishing the levels of GSH and decreasing nitrotyrosine expression and MDA concentration. Together, these results suggest that the protective effect of the HPMC/GSNO solution is at least partially related to its inhibitory effect on oxidative stress. Moreover, other studies have indicated that GSNO decreases the formation of nitrotyrosine and lipid peroxidation in blood, increasing the reduced GSH/oxidized GSH (GSH/GS-SG) ratio in the brain [39–41].

Considering that alveolar bone loss is a severe consequence of periodontal disease, we investigated the role of GSNO on bone regulatory RANK/RANKL/OPG pathway in association with inflammatory and oxidative factors was investigated. It has been demonstrated that TNF-α can lead to bone destruction by acting direct on osteoclast differentiation [42]. TNF-α acts together with IL-1 and RANKL, the receptor activator of nuclear factor-κB ligand, which are abundant in inflammation sites associated with bone destruction, via osteoclast development and activation [43]. Osteoprotegerin (OPG), expressed by many types of cells as osteoblasts, counterregulates the excessive bone loss by antagonizing the RANKL-binding to its receptor RANK [44]. Osteoprotegerin (OPG) and receptor activator of nuclear factor-κB ligand (RANKL) ratio is a key factor to control osteoclast activity and bone resorption [45]. Our results revealed an enhanced expression of RANK in the periodontal tissues of rats submitted to EPD, mainly in osteoclasts, associated with a decrease in the OPG expression and a significant increase in the RANK mRNA levels, as well as in the ratio RANKL/OPG mRNA levels, in the gingival tissue of animals submitted to EPD (HPMC solution group). The topical application of HPMC/GSNO 10 mM solution was able to reduce the RANK expression and increase the OPG expression in the periodontal tissue of the animals. In addition, the topical application of the HPMC/GSNO 10 mM solution led to a significant decrease in the RANK and RANKL/OPG mRNA levels in the gingival tissue, compared with the control HPMC solution group. In summary, our results provide evidence that the topical application of HPMC/GSNO 10 mM solution is capable of reducing alveolar bone loss, pro-inflammatory cytokines production and oxidative stress in an experimental model of periodontal disease, representing a new potential treatment for periodontal diseases.
