# Correction: Topical HPMC/S-Nitrosoglutathione Solution Decreases Inflammation and Bone Resorption in Experimental Periodontal Disease in Rats

**DOI:** 10.1371/journal.pone.0156356

**Published:** 2016-05-19

**Authors:** Conceição S. Martins, Renata F. C. Leitão, Deiziane V. S. Costa, Iracema M. Melo, Glaylton S. Santos, Vilma Lima, Victor Baldim, Deysi V. T. Wong, Luana E. Bonfim, Cíntia B. Melo, Marcelo G. de Oliveira, Gerly A. C. Brito

Fig 9 was incorrectly duplicated as Figs 8 and 9. Please see the corrected Fig 8 here.

**Fig 8:**
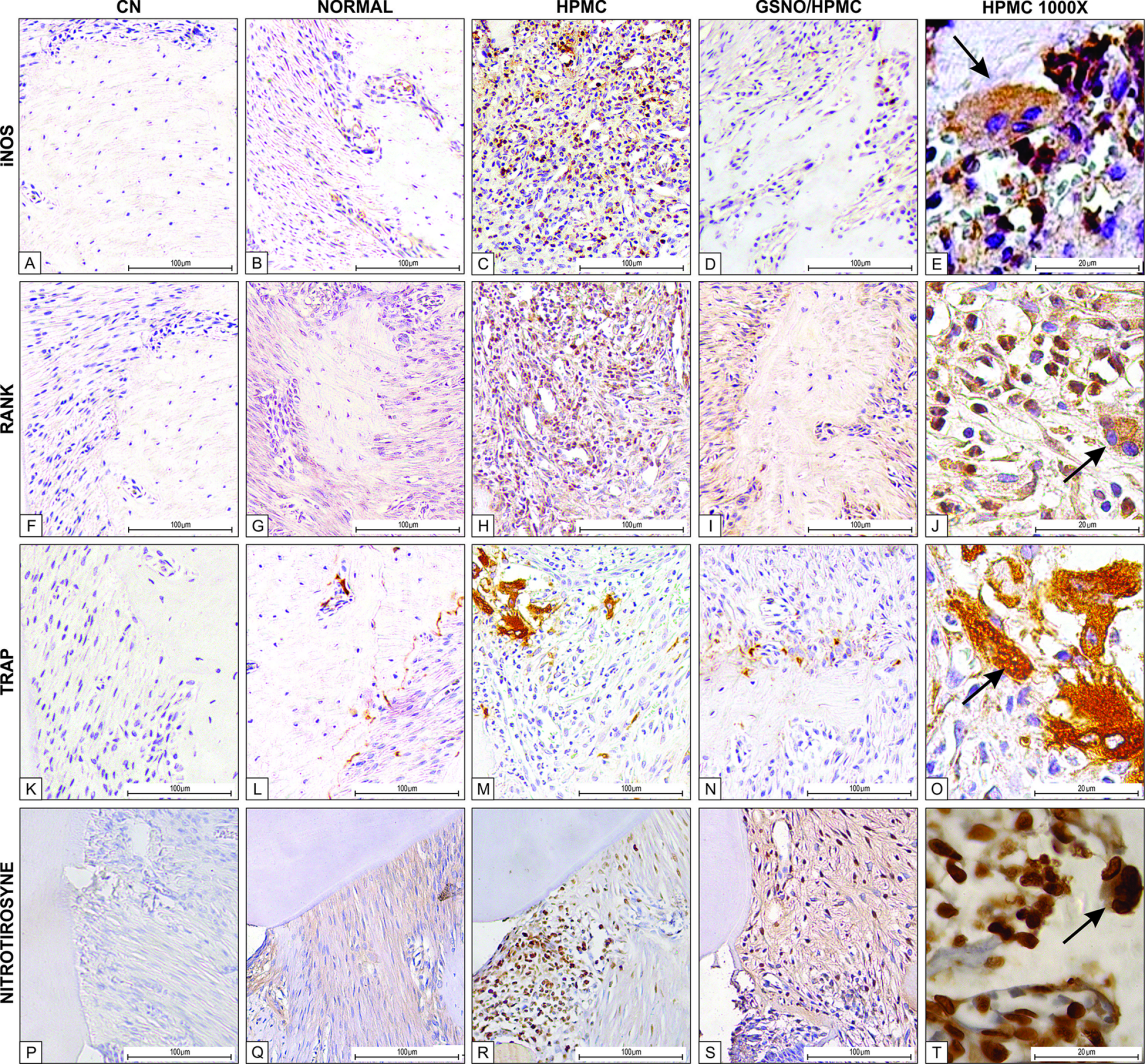
Representative examples of iNOS (1^st^ row), RANK (2^nd^ row) and TRAP (3^rd^ row) immunostaining in experimental periodontitis in rats. Staining was performed using periodontal tissues from normal control animals (b, g, l, q), animals subjected to experimental periodontitis that received topical applications of HPMC (c, h, m, r) or 10 mM HPMC/GSNO (d, i, n, s). Negative controls were samples of periodontal tissue where the primary antibody was replaced with PBS-BSA (5%); no immunostaining was detected (a, f, k, p). Magnification x200. Arrows points to immunostaining osteoclasts in the periodontal tissue of the control HPMC solution group (Magnification x1000).
